# Pure SARS-CoV-2 related AVDS (Acute Vascular Distress Syndrome)

**DOI:** 10.1186/s12879-021-05805-5

**Published:** 2021-01-28

**Authors:** Vincent Jounieaux, Damien Basille, Osama Abou-Arab, Marie-Pierre Guillaumont, Claire Andrejak, Yazine Mahjoub, Daniel Oscar Rodenstein

**Affiliations:** 1 Pneumology Department, University Hospital Centre, Amiens, France; 2 Cardiac, Thoracic-vascular and Respiratory Intensive Care Unit, Department of Anesthesia and Critical Care, University Hospital Centre, Amiens, France; 3 Transversal Cardiology Department, University Hospital Centre, Amiens, France; 4 Pneumology Department, Cliniques Universitaires Saint-Luc, Université Catholique de Louvain, Brussels, Belgium

**Keywords:** SARS-CoV-2, Intrapulmonary shunt, Contrast-enhanced echocardiography

## Abstract

**Background:**

SARS-CoV-2 virus which targets the pulmonary vasculature is supposed to induce an intrapulmonary right to left shunt with an increased pulmonary blood flow. Such vascular injury is difficult to observe because it is hidden by the concomitant lung injury. We report here what may be, to the best of our knowledge, the first case of a pure Covid-19 related Acute Vascular Distress Syndrome (AVDS).

**Case presentation:**

A 43-year-old physician, tested positive for Covid-19, was addressed to the emergency unit for severe dyspnoea and dizziness. Explorations were non informative with only a doubt regarding a sub-segmental pulmonary embolism (no ground-glass lesions or consolidations related to Covid-19 disease). Dyspnoea persisted despite anticoagulation therapy and normal pulmonary function tests. Contrast-enhanced transthoracic echocardiography was performed which revealed a moderate late right-to-left shunt.

**Conclusions:**

This case report highlights the crucial importance of the vascular component of the viral disease. The intrapulmonary shunt induced by Covid-19 which remains unrecognized because generally hidden by the concomitant lung injury, can persist for a long time. Contrast-enhanced transthoracic echocardiography is the most appropriate test to propose in case of persistent dyspnoea in Covid-19 patients.

**Supplementary Information:**

The online version contains supplementary material available at 10.1186/s12879-021-05805-5.

## “Take-away” lessons

- SARS-CoV-2 infection is a vascular disease which is hidden by the lung injury.
- The vascular disorder induced by Covid-19 can persist for a long time.
- Contrast-enhanced transthoracic echocardiography is worth performing even after the Covid-19 infection.

## Background

In Covid-19 infected patients, extraordinarily low blood-oxygen levels have been observed whereas patients described themselves as comfortable [1]. We have suggested that this curious so-called “happy hypoxia” was related to the presence of an intrapulmonary shunt leading to a hypocapnic ventilatory inhibition [2, 3]. In its typical presentation, the parenchymal lung Covid-19 related consolidations hide the intrapulmonary shunt. We report here the first case of a Covid-19 patient in whom the intrapulmonary shunt was unveiled by the absence of concomitant parenchymal lung lesions.

## Case presentation

**Fig. 1:**
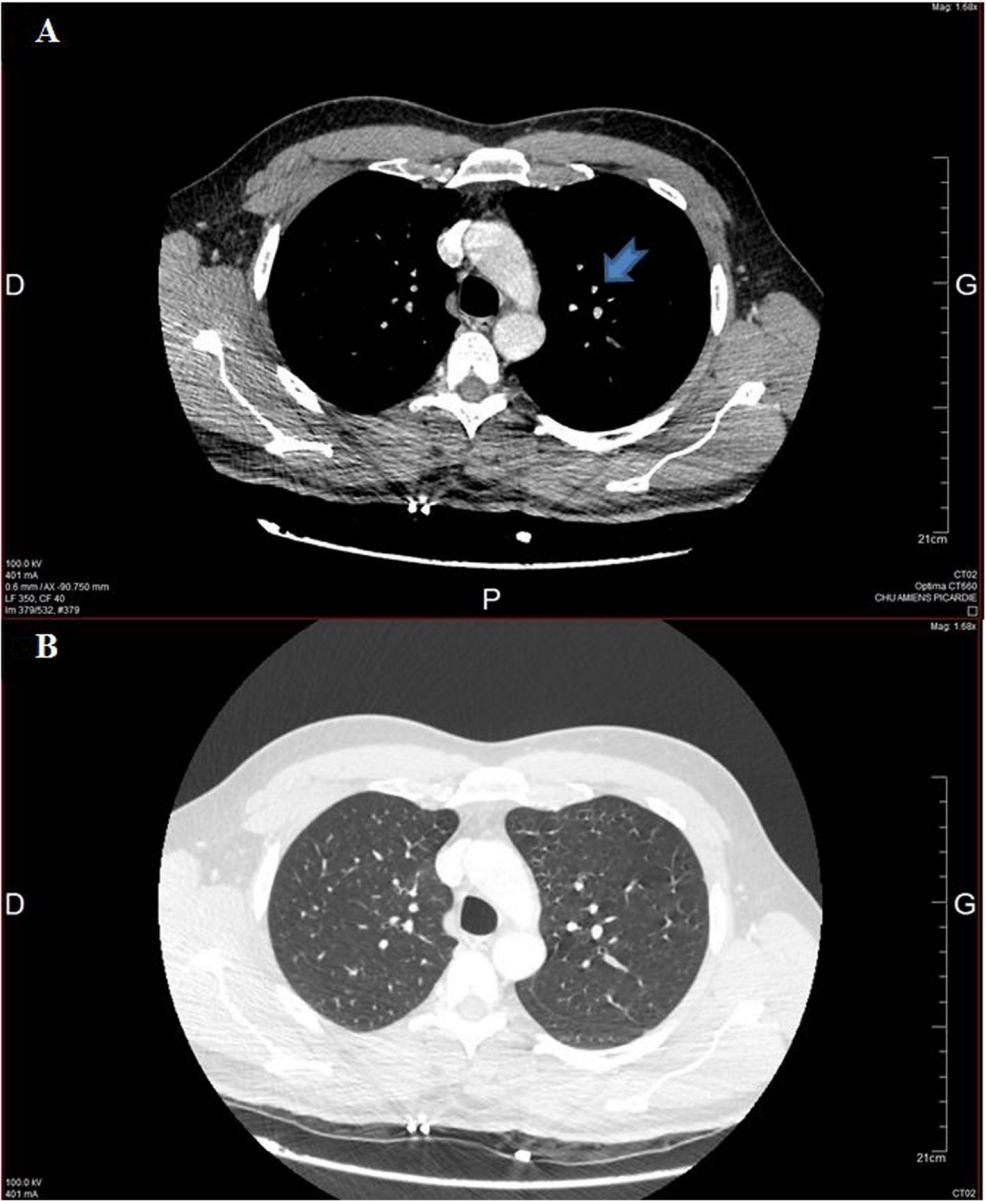
Transverse section CT scan. **a**: Mediastinal window showing a possible left superior lobar sub-segmental defect (arrow), subject to a poor quality of the contrast injection. **b**: Corresponding parenchymal window. Note the absence of lung parenchymal consolidation or ground-glass opacities

**Fig. 2:**
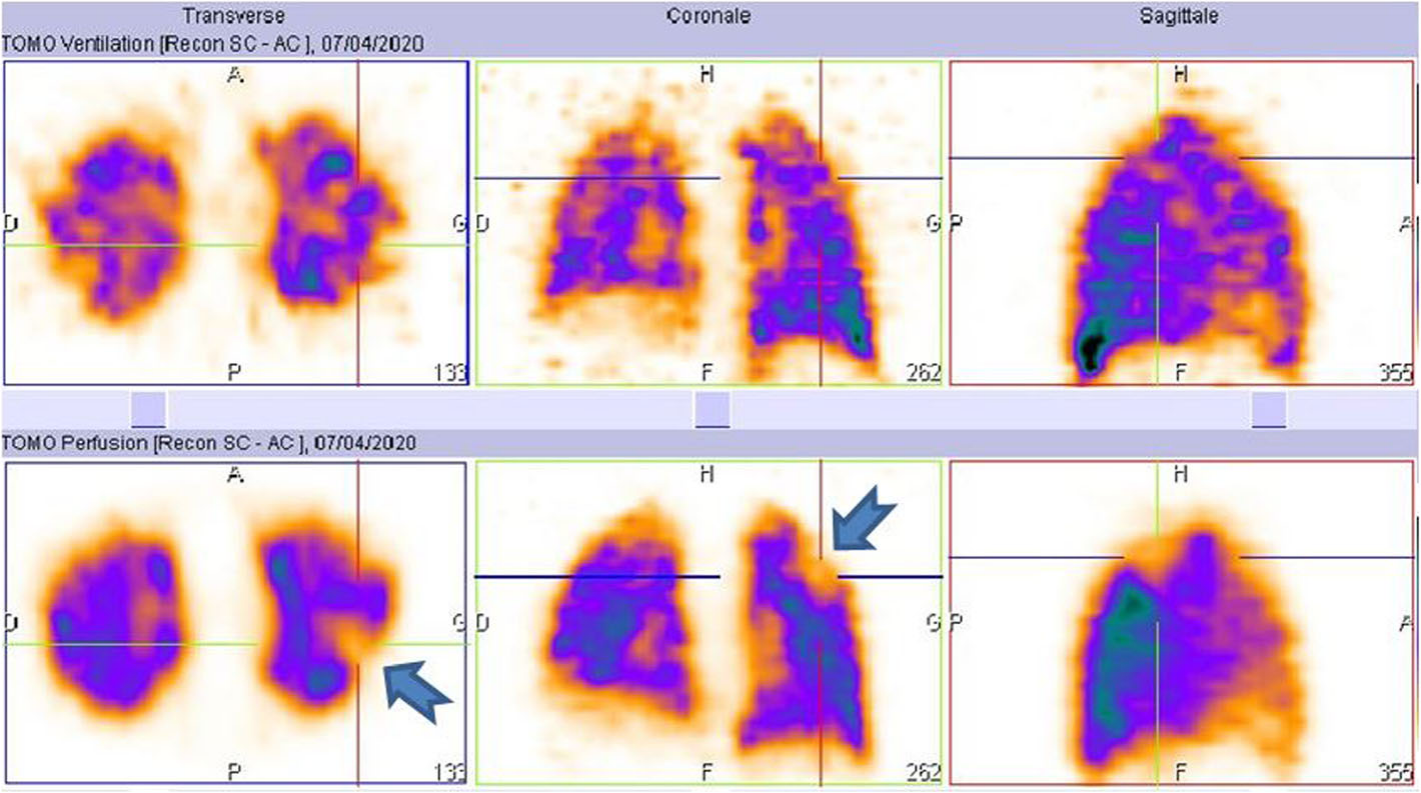
Transverse, sagittal and coronal sections of ventilation (upper panel) and perfusion (lower panel) scanning showing a left superior lobar sub-segmental mismatch (arrow)

A 43-year-old physician presented to the emergency department because of severe dyspnoea and dizziness without loss of consciousness. This malaise started after returning home by bicycle and was accompanied by paraesthesia of jaw and arms. The patient described his dyspnoea as a frightening air thirst with end-inspiratory blockade and rated it at 8 on a scale of 0 to 10, with 10 indicating the most severe dyspnoea. No chest pain was noted but a feeling of imminent death with impairment to speak. This athletic patient (1.80 m, 84 Kg) has no comorbidities, takes no medication, has no history of a clotting disorder, and there is no family history of coagulopathy. Ten days before presentation, the patient had been tested positive for Covid-19 (due to a professional or familial exposure to SARS-CoV-2 as his wife and son developed a Covid-19 infection before him), after he felt dry cough, shortness of breath (2/10), fatigue and diarrhoea (no fever and anosmia). At admission, the patient’s temperature was 36.6 °C, blood pressure 115/60 mmHg, pulse 68 beats per minute and respiratory rate 10 breaths per minute; a pulse oximeter reading indicated that his oxygen saturation was 100% while he was breathing ambient air. The lungs were clear, and the results of the physical examination were otherwise normal. Electrocardiography showed sinus rhythm with no evidence of right bundle branch block or right ventricular strain. Laboratory testing revealed slightly elevated d-dimer (0.56 μg/mL) and creatinine kinase (231 U/L) levels but no elevation in troponin levels. Severe hypophosphatemia was present (0.23 μmol/L). The arterial blood gas (ABG) levels while breathing room air were as follows: pH, 7.55; PaO_2_, 112 mmHg; and PaCO_2_, 23.8 mmHg. His haemoglobin level was 15 g/dL, white blood cell count was 7.9/mL (with normal lymphocyte count), platelet count was 176,000/mL and CRP was normal (1.5 mg/L). An echocardiography was performed showing normal right and left ventricular functions without any pulmonary arterial hypertension. A computed tomographic (CT) pulmonary angiography showed no evidence of pulmonary embolism (PE) nor pulmonary lesions related to Covid-19 (Fig. 1) and Doppler ultrasound of the legs was normal. However, in the context of Covid-19 outbreak, anticoagulation was started. The patient was given subcutaneous low molecular weight heparin, tinzaparin 15,000 units daily, with a treatment recommendation for 3 months. The following day, a ventilation-perfusion scanning was performed showing only one scarce left superior lobar sub-segmental mismatch (Fig. 2). CT pulmonary angiography was reviewed by two others radiologists who could not exclude a left superior lobar sub-segmental embolism (CT of poor quality due to a contrast injection disturbed by the patient’s deep inspirations). Tinzaparin anticoagulation was maintained, with a periodic platelets count monitoring.

Four weeks later, the patient was addressed to our Pneumology department for persistent dyspnoea, rated 0 in the morning and 6 at the end of the day (on a scale of 0 to 10), and essentially induced by physical activity. No history of hyperventilation syndrome was found and the Nijmegen score was of 0/64. A persistent rhinorrhoea and fatigue were noticed. Physical examination remained unremarkable without any tachypnea but a detailed questioning revealed that the patient usually resorted to short periods of rests lying down on prone position between consultations during his work to alleviate his dyspnoea. Pulmonary function tests and diffusion capacity were normal as well as the exhaled NO (15 ppb). StcO2 at rest was of 96%. Laboratory testing revealed normal levels of D-dimer, fibrinogen and CRP. In the context of Covid-19 outbreak, a contrast-enhanced transthoracic echocardiography with Valsalva manoeuvres was performed showing a moderate late right-to-left shunt (30 microbubbles appearing in the left atrium after ten systoles) (Additional file 1; video). A control of the ventilation-perfusion scanning was found normal. Thus, persistent pulmonary shunt related to a Covid-19 infection was diagnosed 45 days after the initial infection. Thereafter dyspnoea improved slowly and the anticoagulation therapy was proposed to be maintained for a total duration of 3 months.

## Discussion and conclusions

We recently hypothesized that Covid-19 disease is characterized, at any stages of the disease, by an increased pulmonary blood flow with intrapulmonary right to left shunt leading us to introduce the acronym “AVDS” (Acute Vascular Distress Syndrome) [4]. In spontaneously breathing patients hospitalized in ward for Covid-19 pulmonary infection, disproportionate hypoxemia (often with a PaO_2_/FiO_2_ ratio below 300 mmHg) is often observed despite Chest CT scans showing quite limited ground-glass like opacities suggesting a vascular, rather than parenchymal, dysfunction. Vascular abnormalities have recently been described in a patient with COVID-19 pneumonia without evidence of pulmonary embolism but in whom dual-energy CT-scan showed dilated sub-segmental vessels proximal to, and within, ground-glass opacities and consolidations with, on images of pulmonary blood volume, a decreased perfusion within these opacities and a peripheral halo of higher perfusion [5]. Such anatomical lung vascular disorders can explain the hypoxemia observed in these Covid-19 patients through a low ventilation-to-perfusion (VA/Q) ratio where VA is preserved and Q is increased. The originality of our case-report is to describe for the first time the presence of a significant late right to left pulmonary shunt as detected through contrast-enhanced echocardiography in a Covid-19 patient without any lung injury or major PE, supporting the key role of a long-lasting vascular disorder in this new viral infection.

The acute episode experienced by the patient was initially attributed to PE as an hyperventilation was noted on blood gases data with alkalosis, hypocapnia and normoxia associated with a slight increase in D-dimers. In the context of Covid-19 outbreak, such diagnostic appeared relevant as many publications have drawn attention on the increased risk [6]. However, with the actual better knowledge of the Covid-19 disease, the alternative diagnosis of an acute vascular disease related to the Covid-19 infection [4] appears more likely than those of PE, all the more so that the symptomatology, though attenuated, persisted at the time of the demonstration of the late right to left shunt despite normalisation of the ventilation-perfusion scanning.

At presentation the patient was not hypoxemic; his PaO_2_ and StcO_2_ were even above normal values as was his alveolar oxygen partial pressure (PAO_2_) that we have calculated at 120.4 mmHg according to the formula by Kanber et al. [7]. We hypothesize that the pure intrapulmonary shunt due to Covid-19 infection (without pulmonary parenchymal damage) did indeed induce in our healthy and athletic patient a mild initial hypoxemia leading to a compensatory increase in minute ventilation. Because CO_2_ is much more diffusible than O_2_ and, due to the CO_2_ dissociation curve, to a linear relationship between CO_2_ excretion and minute ventilation, hyperventilation would rapidly lead to hypocapnia which is known to be a powerful inhibitor of the ventilation. We have previously shown that below a PaCO_2_ 29.3 mmHg (23.8 mmHg in our patient), deep oxygen desaturations (up to 64%) are unable to elicit an increase in minute ventilation in normal subjects [8]. This extreme hypocapnia may explain many of the patient’s symptoms and laboratory findings noted by the emergency department (dyspnoea, paraesthesia of chin and arms, severe alkalosis despite a low respiratory rate and hypophosphatemia, unusually high StcO_2_ and the increased PAO_2_ and PaO_2_). Moreover, evidence was found in humans for an association between hyperventilation-induced hypocapnia and a reduction in cerebral perfusion leading to syncope defined as transient loss of consciousness [9], explaining the critical threat felt by our patient. In addition, the high values of PAO_2_ might have widened the alveolar-mixed venous blood oxygen partial pressure, increasing the diffusion pressure of oxygen and the pulmonary oxygen uptake (as has been suggested when apneas repeat in rapid succession [10]. If we add that the alkalosis would increase the avidity of haemoglobin for oxygen, the absence of hypoxemia at presentation may be accounted for. Such situation is rare during the Covid-19 infection as alveolar damage is usually present; leading to an alteration of the alveolar ventilation (VA) that joined to the high pulmonary perfusion aggravates the low ventilation-to-perfusion (VA/Q) ratio. So, in its typical presentation, the parenchymal lung Covid-19 related consolidation limits hypocapnia, aggravates hypoxemia and hides the presence of a vascular component in the form of intrapulmonary shunting. The benefit of rest periods in prone position on dyspnoea reported by this patient is not surprising. Prone-positioning modifies lung pulmonary blood flow, increasing it in the anterior part of the thorax compared to supine position. Because lung shape is conical, prone position decreases global intra-pulmonary shunt by decreasing blood flow in the larger part (posterior) of the lungs. This decrease in blood flow increases VA/Q ratio and thereby oxygenation.

Finally, the association of the hypoxia caused by the intrapulmonary shunt and the subsequent hypocapnic ventilatory inhibition may account for this curious form of dyspnoea described by some patients (air thirst, end-inspiratory limitation with a low or normal respiratory frequency), or even for the absence of dyspnoea in the presence of mild to severe hypoxemia [3, 4]. This type of dyspnoea could be related in part to deventilation dyspnoea which is observed in patients with severe COPD immediately after interruption of nocturnal non-invasive ventilation (NIV) and preventing the patient from getting out of bed and carrying out usual daily activities for more than 30 min after one night of NIV [11].

Today, we do not precisely know the underlying mechanisms leading to Covid-19 vascular injury and hypotheses can only be proposed. The intra pulmonary shunt may be related, as seen in the hepatopulmonary syndrome [12], to a diffuse dilatation of the pulmonary precapillary and capillary vessels and/or to pleural and pulmonary arteriovenous communications. Pathological studies focusing on lung vasculature may shed light on the mechanisms of intrapulmonary shunt. Because SARS-CoV-2 has been shown to establish itself in the host through angiotensin-converting enzyme 2 (ACE2) cellular receptor [13], the presence of ACE2 receptors on vascular endothelial cells might explain the major role of the vascular component in Covid-19 pneumonia. Moreover, it has been shown that the loss in ACE2 may promote vascular dysfunction with endothelial activation of coagulation, overexpression of nitric oxide and inflammation through a possible down regulation in ACE 2 by a cleavage effect through a protease called ADAM 17 [14]. Symptoms in Covid-19 infection may be also explained through an acute vasculitis [15] which probably will not be limited to lungs but will concern other organs as numerous extrapulmonary Covid-19 lesions have been described or suggested in the literature [16–19].

From all these particular aspects observed in our patient, we dare suggest this is the first observation of a pure AVDS without lung parenchymal infiltration related to SARS-CoV-2 infection.

## Supplementary Information

**Additional file 1.** Video of the contrast-enhanced transthoracic echocardiography showing a moderate late right-to-left shunt (30 microbubbles appearing in the left atrium after ten systoles).

## Data Availability

All data generated or analysed during this study are included in the manuscript.

## Abbreviations

ABG: arterial blood gas
ACE2: angiotensin-converting enzyme 2
AVDS: Acute Vascular Distress Syndrome
COPD: chronic obstructive pulmonary disease
CT: computed tomographic
FiO_2_: fraction of inspired oxygen
NIV: non-invasive ventilation
PAO_2_: alveolar oxygen partial pressure
PaO_2_: arterial oxygen partial pressure
PaCO_2_: arterial carbon dioxide partial pressure
PE: pulmonary embolism
StcO_2_: transcutaneous arterial oxygen saturation
VA: alveolar ventilation
VA/Q: ventilation-to-perfusion
